# Prichard and Symonds in Especial Relation to Mental Science, with Chapters on Moral Insanity

**Published:** 1891-12

**Authors:** 


*Prichard and Symonds in especial Relation to Mental Science, with Chapters on Moral Insanity.* By D. HACK TUKE, M.D., LL.D. Pp. 116. London: J. & A. CHURCHILL. 1891.

Dr. Hack Tuke, in his memoir of Dr. Prichard and Dr. Symonds, does not attempt to give an exhaustive account of the work of either of these distinguished physicians. He speaks of course of Dr. Prichard's renown as a physician, and as an ethnologist, and bears testimony to his merits as the philologist who first proved the position of the Keltic languages as a branch of the Indo-European.

But since his *brochure* treats of moral insanity, the author is mainly concerned with the views held by these physicians on mental disease, or on subjects bordering on mental pathology. He selects for notice from Dr. Symonds' numerous writings the essays on the relations between the mind and muscles, on sleep and dreams, on apparitions, habit, the principles of beauty, and on criminal responsibility in relation to insanity.

Dr. Prichard became one of the first Commissioners in Lunacy, and even if he had not been so remarkable in other departments of knowledge, his name would always have been distinguished by the views he advocated on moral insanity. The term, objected to, as it is, by jurists, is the expression of a condition almost universally recognised by alienist physicians. Its important factor is not loss of memory, not delusion or hallucination, not any deficiency of talent or genius, not any lack of mental acuteness, and certainly no incoherence of ideas or language, but a deficiency or impairment of moral feeling or self-control, such being either the development of a character natural to the individual, or a departure from it, which contrasts most strikingly with its former traits. Dr. Prichard initiated this view, and Dr. Symonds records cases of it within his own experience. In its later development it is frequently associated with intellectual decadence, and in some cases it ends in dementia; but for years the patient may show, to ordinary observers, nothing but symptoms that own no broad line of demarcation from moral turpitude; although a very careful examination will often detect some intellectual disorder. Dr. Yellowlees believes that it is invariably the result of hereditary tendency, and that it is associated with other forms of neurotic disorder among the relatives. Dr. Tuke has done good service in again calling attention to this form of disease in connection with the names of two of our greatest Bristol medical worthies.

